# A Question for Boards of Guardians

**Published:** 1903-01-24

**Authors:** 


### A QUESTION FOR BOARDS OF GUARDIANS.

Mr. John James Jones, of Sandgate, to whose proceedings *Truth* has, in the public interest, persistently called attention for years, has lately devoted his attention to Boards of Guardians, to whom he has written offering to receive phthisical cases for weekly payments. Mr. John James Jones claims that all such Poor Law cases will receive at his Homes the benefit of the Nordrach treatment. We had reason to fear that this claim on Mr. John James Jones's part might be pitched a good deal too high, and in the interests of the poor we thought it desirable to request two members of our staff to place themselves in communication with Mr. John James Jones, with a view to make an appointment with him to inspect his "Homes" at Sandgate. The answer which our representatives received is signed "John James Jones," dated January 7th, 1903, and is as follows :—

"Replying to yours of yesterday's date, I much prefer your not visiting my establishments at the present time. I visited in September and October last 23 sanatoria in Germany and Switzerland. I also visited several consumptive hospitals and sanatoria in England during the past year, and am now having alterations and additions made which will enable me to carry out, under medical supervision, the 'open-air treatment' in a more efficient manner.

"Pardon me for calling your attention to the fact that these institutions are not in any way maintained by public contributions, but are conducted on strictly business principles."

It is interesting to note that Mr. John James Jones inferentially throws over the claim put forward in his circular to the Guardians, that pauper patients sent to him will have the advantage of the Nordrach treatment, as he now confesses that he is having alterations and additions made which will enable him to carry it out. It will further be observed that he maintains that his institutions "are conducted on strictly business principles," a fact which we should be the last to dispute, knowing Mr. John James Jones's history. But we would point out to him, and to the Guardians all over the country, that, as a matter of fact, Mr. John James Jones states in his papers that he will take patients who have a subscriber's letter at a reduced rate of payment, a fact which he apparently forgot when informing us that these institutions "are not in any way maintained by public contributions." We have heard of several patients who have left the Homes in disgust, and we feel it to be a public duty to call the attention of the Guardians of the Poor all over the country to the necessity for exercising extreme caution before they enter into any contract with or send any cases to Mr. John James Jones, in view of the facts stated in his letter, or at all, until he is prepared to submit his establishments to expert investigation, so that they may have independent testimony to guide them to a decision as to whether or not it is just or defensible to entrust serious cases to the care of Mr. John James Jones of Sandgate.

